# Defining the Roles of the Cation Diffusion Facilitators in Fe^2+^/Zn^2+^ Homeostasis and Establishment of Their Participation in Virulence in *Pseudomonas aeruginosa*

**DOI:** 10.3389/fcimb.2017.00084

**Published:** 2017-03-20

**Authors:** Agostina Salusso, Daniel Raimunda

**Affiliations:** Consejo Nacional de Investigaciones Científicas y Técnicas, Instituto de Investigación Médica Mercedes y Martín Ferreyra, Universidad Nacional de CórdobaCórdoba, Argentina

**Keywords:** cation diffusion facilitator, transition metal homeostasis, efflux, iron, zinc, virulence

## Abstract

Transporters of the cation diffusion facilitator (CDF) family form dimers that export transition metals from the cytosol. The opportunistic pathogen *Pseudomonas aeruginosa* encodes three homologous CDF genes, *czcD* (PA0397), *aitP* (PA1297), and *yiiP* (PA3963). The three proteins are required for virulence in a plant host model. Disruption of the *aitP* gene leads to higher Fe^2+^ and Co^2+^ sensitivity together with an intracellular accumulation of these ions and to a decreased survival in presence of H_2_O_2_. Strains lacking *czcD* and *yiiP* showed low Zn^2+^ sensitivity. However, in iron-rich media and in the presence of Zn^2+^ these strains secreted higher levels of the iron chelator pyoverdine. Disruption of *czcD* and *yiiP* in a non-pyoverdine producer strain and lacking the Zn^2+^-transporting ATPase, increased the Zn^2+^ sensitivity and the accumulation of this ion. Most importantly, independent of the pyoverdine production strains lacking CzcD or YiiP, presented lower resistance to imipenem, ciprofloxacin, chloramphenicol, and gentamicin. These observations correlated with a lower survival rate upon EDTA-lysozyme treatment and overexpression of OprN and OprD porins. We hypothesize that while AitP is an Fe^2+^/Co^2+^ efflux transporter required for Fe^2+^ homeostasis, and ultimately redox stress handling, CzcD, and YiiP export Zn^2+^ to the periplasm for proper Zn^2+^-dependent signaling regulating outer membrane stability and therefore antibiotic tolerance.

## Introduction

Transition metals (TM) participate in vital physiological processes such as respiration, reactive oxygen species handling, and transcriptional regulation (Fraústo Da Silva and Williams, 2001). During infection processes, bacteria face host-driven changes in TM bioavailability, leading to their scarcity or abundance, both conditions detrimental for the bacteria. An excess of these nutrients leads to cellular toxicity, through mechanisms involving the displacement of cognate metals from catalytic and structural sites of metalloproteins (Ranquet et al., 2007), or by producing reactive oxygen species through Fenton-like reactions (Macomber and Imlay, 2009; Imlay, 2014). On the other hand, metal scarcity requires a highly regulated metabolic machinery to ensure metalloprotein synthesis (Argüello et al., 2013; Kehl-Fie et al., 2013). A large number of membrane transporter families participate specifically in metal efflux or uptake in order to maintain each individual TM quota and satisfy the metabolic demand.

The cation diffusion facilitator (CDF) family participates in TM efflux from the cytosol to the periplasm (Nies, 2003). TM efflux is coupled to H^+^ entry and thus is driven by the electrochemical H^+^-gradient across the bacterial plasma membrane (Guffanti et al., 2002; Gupta et al., 2014). Members of the family present six transmembrane segments (TMS) and a cytosolic C-terminal domain (CTD) involved in transport regulation and protein dimerization. In most cases, metal transport is accomplished by a homodimeric structure, although, in organisms bearing several CDF paralogs, molecular and phenotypic evidence suggests that functional heterodimerization occurs (Uebe et al., 2011). The crystal structure of the Zn^2+^ transporting *E. coli* member YiiP revealed three metal binding sites (A, B, and C; Lu et al., 2009). Site A is formed by residues located in TMS 2 and TMS 5 and is indispensable for transport, while sites B and C seem to be unrelated to this process. However, site C is involved in allosteric regulation by coupling the sensing of cytoplasmic Zn^2+^ to the binding of the ion at site A.

Cellular roles for CDF transporters have mostly been linked to transition metal tolerance, i.e., exporting metal surplus from the cell to avoid excessive accumulation and toxicity. In *Streptococcus penumoniae*, virulence is reduced when the function of the CDF transporter, MntE, is abolished (Rosch et al., 2009). The mutant strain accumulates Mn^2+^ specifically when grown in the presence of this ion. Similarly, in *Sinorhizobium meliloti*, the CDF YiiP plays a role in Mn^2+^ efflux and plants infected with this strain grow poorly due to a lesser infection capacity of the bacteria (Raimunda and Elso-Berberian, 2014). In humans, mutations in the gene coding for the ortholog ZnT10 lead to Parkinsonism with concomitant hypermanganesemia (Quadri et al., 2012).

More than half of whole genome-sequenced bacteria have two or more CDF paralog genes encoded in their genome (Ren et al., 2007). In these organisms, it is probable that different CDF proteins transport alternative substrates. Alternatively, the presence of CDF transporters sharing similar substrate specificity with members of other families in one organism opens the possibility to inquire about the different roles played among these, or support functional redundancy.

*P. aeruginosa* PAO1 has three uncharacterized CDF transporters in loci PA0397, PA1297, and PA3963. Two of them, PA0397 and PA3963, share high sequence similarity to the Zn^2+^-transporting *E. coli* member YiiP and thus a role in Zn^2+^ metal tolerance can be inferred. However, existing evidence indicating that the *P. aeruginosa* Zn^2+^-ATPase ZntA participates in the ion tolerance (M. González-Guerrero personal communication), argues against this possibility. PA1297 presents a poly-his stretch between TMS 4 and TMS 5 and belongs to the Zn^2+^ transporting sub-class (Montanini et al., 2007) although recent evidence supports also a role in Co^2+^ homeostasis (Cubillas et al., 2013). The absence of an Fe^2+^/Co^2+^ transporting ATPase in *P. aeruginosa* PAO1 suggests that PA1297 product may play a role in Fe^2+^/Co^2+^ homeostasis.

In this study we evaluated the specificity of transport, the cellular roles and the participation in infection of the *P. aeruginosa* CDF members. Our data suggest that they all are required for proper virulence and that this is linked to their roles in export of transition metals. One member, AitP (Alternative iron transport protein, PA1297), exports Fe^2+^/Co^2+^, while CzcD (PA0397) and YiiP (PA3963) are involved in Zn^2+^ efflux. Importantly, Fe^2+^ transport through AitP protects against redox stress, while Zn^2+^ transport through CzcD and YiiP is coupled to outer membrane permeability.

## Materials and methods

### Bacterial strains and growth conditions

*Pseudomonas aeruginosa* PAO1 wild-type strain, *czcD::dTn5* (PW1733/PA0397 insertional mutant), *aitP::dTn5* (PW3352/PA1297 insertional mutant) and *yiiP::dTn5* (PW7707 /PA3963 insertional mutant) were obtained from the Comprehensive *P. aeruginosa* Transposon Mutant Library at the University of Washington Genome Center (Jacobs et al., 2003; Table 1). Strains lacking pyoverdine were generated from parental strain *P. aeruginosa* PAO1, *czcD::dTn5* and *yiiP::dTn5* by insertional mutation. An internal fragment corresponding to 600 bp of the gene PA2386/*pvdA* was cloned into pCHESIΩ-Km, between EcoRI and BamHI sites (Llamas et al., 2003). Subsequently, the plasmid was mobilized from *E. coli* DH5α cells into *P. aeruginosa* by triparental conjugation using *E. coli* HB101 (pRK2013) strain as helper. The resulting strains SM010 (*pvdA* mutant), DM011 (*pvdA* and *czcD* mutant) and DM013 (*pvdA* and *yiiP* mutant) were used to generate the mutants lacking the Zn^2+^ transporting ATPase, ZntA. An internal fragment corresponding to 450 bp of the gene PA3690/*zntA* was cloned into pJQ200SK-Gm (Quandt and Hynes, 1993) and mobilized into *P. aeruginosa* mutant strains as described above resulting in DM110 (*zntA* and *pvdA* mutant), TM111 (*zntA, pvdA*, and *czcD* mutant) and TM113 (*zntA, pvdA*, and *yiiP* mutant; Table 1). Complemented strains were obtained by cloning each CDF plus 500 bp of the putative promoter region in pUC18T-mini-Tn7T-Gm (Choi and Schweizer, 2006). Since *yiiP* is predicted to be part of a polycistronic operon, the 500 bp upstream region of the contiguous loci PA3962 was cloned and joined to *yiiP*. The megaprimer-PCR method was employed to obtain the fusion (Sambrook et al., 1989). The primers used in this and other procedures are listed in Table 1. The resulting vectors were inserted in the genome of *P. aeruginosa* by tetraparental mating conjugation method as described (Choi and Schweizer, 2006). Transconjugants were selected on selective Luria–Bertani (LB) agar plates. All constructs and mutants were confirmed by DNA sequencing. Cells were grown at 37°C in LB or Müller-Hinton media as indicated, supplemented with tetracycline (30–60 μg/ml), gentamicin (30 μg/ml), and kanamycin (300 μg/ml) as required.

**Table 1:** **Strains and primers used in this study**.

**Strain/primer**	**Relevant characteristics/sequence**	**References/Use**
***Pseudomonas aeruginosa***		
PAO1	Wild-type	
*czcD::dTn5*	Tn5 derivative ISlacZ/hah insertion in *czcD* (PA0397), Tc^r^	Jacobs et al. (2003)
*aitP::dTn5*	Tn5 derivative ISphoA/hah insertion in *aitP* (PA1297), Tc^r^	Jacobs et al. (2003)
*yiiP::dTn5*	Tn5 derivative ISphoA/hah insertion in *yiiP* (PA3963), Tc^r^	Jacobs et al. (2003)
SM010	*pvdA::*pCHESIΩ-Km; Km^r^	This study
DM011	Tn5 derivative ISlacZ/hah insertion in *czcD, pvdA::*pCHESIΩ-Km, Tc^r^ Km^r^	This study
DM013	Tn5 derivative ISphoA/hah insertion in *yiiP, pvdA::*pCHESIΩ-Km, Tc^r^ Km^r^	This study
DM110	*pvdA*::pCHESIΩ-Km, *zntA::*pJQ200SK-Gm, Km^r^ Gm^r^	This study
TM111	Tn5 derivative ISlacZ/hah insertion in *czcD, pvdA::*pCHESIΩ-Km, *zntA::*pJQ200SK-Gm, Tc^r^ Km^r^ Gm^r^	This study
TM113	Tn5 derivative ISphoA/hah insertion in *yiiP, pvdA::*pCHESIΩ-Km, *zntA::*pJQ200SK-Gm, Tc^r^ Km^r^ Gm^r^	This study
C-*czcD*	Tn5 derivative ISlacZ/hah insertion in *czcD, att*Tn7*::*mini-Tn7T-P_czcD_-*czcD*, Tc^r^ Gm^r^	This study
C-*aitP*	Tn5 derivative ISphoA/hah insertion in *aitP, att*Tn7*::* mini-Tn7T-P_aitP_-*aitP*, Tc^r^ Gm^r^	This study
C-*yiiP*	Tn5 derivative ISphoA/hah insertion in *yiiP, att*Tn7*::* mini-Tn7T-P_yiiP_-*yiiP*, Tc^r^ Gm^r^	This study
***Escherichia coli***		
HB101	Helper strain for *P. aeruginosa* transformation	Llamas et al. (2003)
DH5α	*supE44 ΔlacU169* (φ80 *lacZ* ΔM15) *hsdR17 recA1 endA1 gyrA96 thi1 relA1*	Llamas et al. (2003)
**Primers**		
For-DM-pvdA	ATCGGAATTCCGCTGGCACGGCAACACCCTG	Clonning internal *pvdA* fragment into pCHESI
Rev-DM-pvdA	CGATGGATCCCTATCGTCCGCCGGCTTGAGC	Clonning internal *pvdA* fragment into pCHESI
For-DM-zntA	ATCGGAATTCCTGGATGCCGGCGAAAATAC	Clonning internal *zntA* fragment into pJQ200SK
Rev-DM-zntA	CGATGGATCCGCTTCCAGTTCCACTTGCTT	Clonning internal *zntA* fragment into pJQ200SK
For-Prom0397	AGTCGGTACCGTAGAGCACGCCGAGGAAG	Full length *czcD* plus 500 bp upstream
Rev-stop0397	ACGTCCCGGGTCAGTAGGCCAGCGGCTC	Full length *czcD* plus 500 bp upstream
For-Prom1297	AGTCGGTACCCGTCGCACAGCGACTCC	Full length *aitP* plus 500 bp upstream
Rev-stop1297	AAGCTTTCAGGCGGCGAGCGGGA	Full length *aitP* plus 500 bp upstream
For-Prom3963	AGTCGGTACCCAGGCCGACACCGTCCA	500 bp upstream PA3962
Rev-Prom3963	GGGAATCGGGACTCATGTATGACCTCCGGGTGAAC	500 bp upstream PA3962
For-3963	GTTCACCCGGAGGTCATACATGAGTCCCGATTCCC	Full length *yiiP*
Rev-stop3963	AAGCTTTCAACTGACGGTTTCCTTGC	Full length *yiiP*

### Bioinformatics analyses

**Figure 1:**
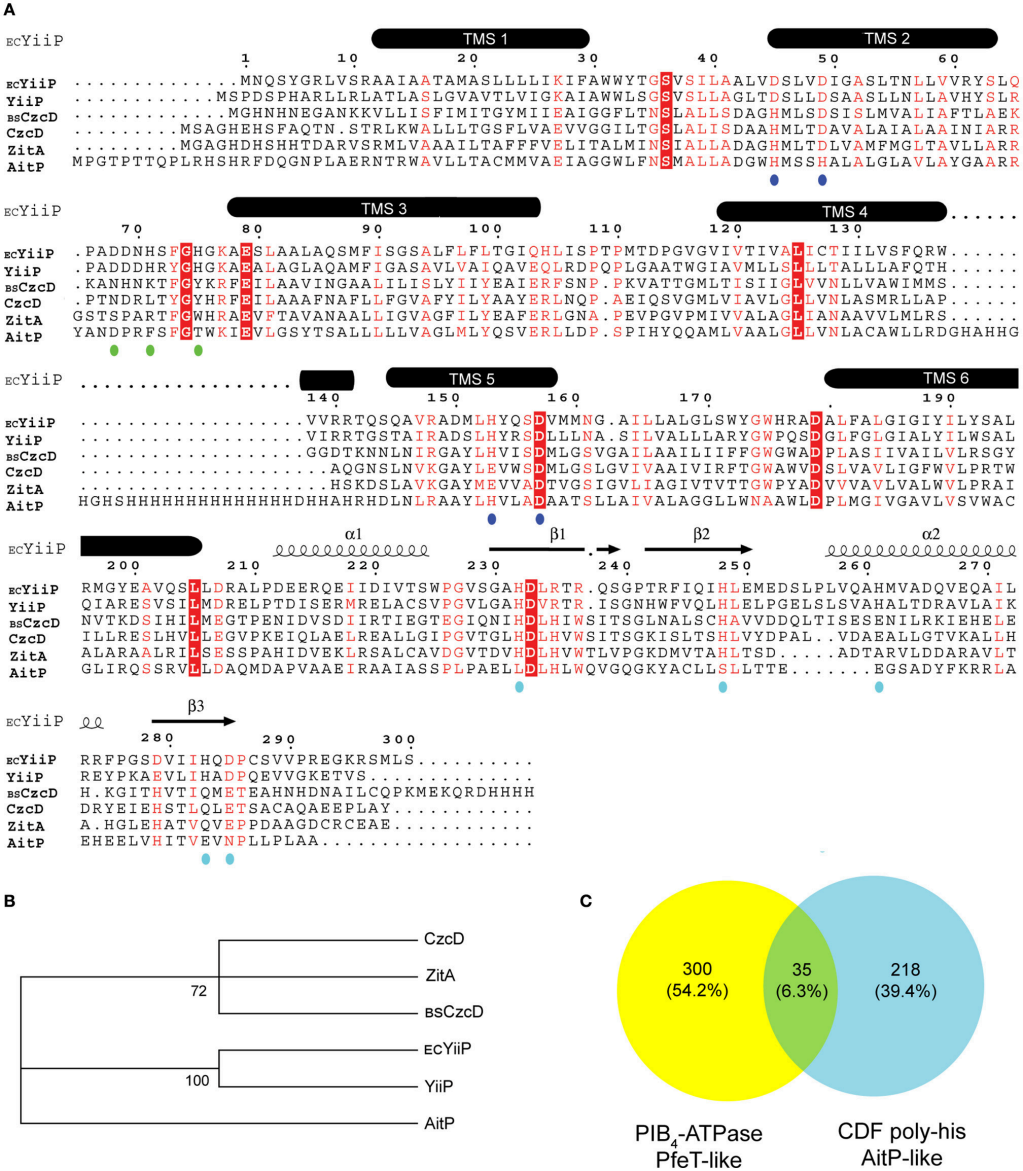
**Multiple protein sequence alignment of CDF members in *Pseudomonas aeruginosa* PAO1 with previously characterized homologous members and analysis of the presence of Fe^2+^ exporters in reference bacterial genomes**. Organisms and corresponding loci/protein names used are: *P. aeruginosa* PAO1, PA3963/YiiP, PA0397/CzcD, and PA1297/AitP; *Escherichia coli* K12-MG1655, *fieF*/_EC_YiiP; *Bacillus subtilis* 168, *czcD*/_BS_CzcD; *Mycobacterium smegmatis* mc^2^155, MSMEG_0755/ZitA. TMS, transmembrane segments. Putative residues in metal binding sites A, B, and C are indicated with blue, green, and cyan dots, respectively. Conserved residues are shown in white letters and red boxes. Red letters indicate conservative substitution. *E. coli* YiiP 3D structure PDB 3H90 was used to depict TMS **(A)**. Molecular phylogenetic analysis by Maximum Likelihood method is shown. The percentage of trees in which the associated sequences clustered together is shown next to the branches **(B)**. Venn diagram depicting the number of reference bacterial genomes having a P_IB4_-ATPase (yellow), a CDF poly-his (cyan), or both (green) **(C)**.

All sequences in the study were retrieved from the NCBI database. Alignments were performed in MUSCLE (Edgar, 2004), and analyzed with ESPript software (Gouet et al., 1999). *E. coli* YiiP, *B. subtilis* CzcD, and *M. smegmatis* ZitA were included in the alignment for comparison. The proteins that were not on KEGG were named with a two-letter key to denote their organismal origin, as indicated in Figure 1. Evolutionary analyses were conducted in MEGA6 (Tamura et al., 2013). Searches for poly-his CDF and Fe^2+^/Co^2+^-ATPases were performed in BlastP (PHI-Blast) using as query the protein sequence of *P. aeruginosa* AitP (accession number NP_249988.1) and *M. semgmatis* CtpD (accession number AFP41699.1) and against bacterial reference genomes database. Searches were oriented with H-H-H-[HD] or H-E-G-[GS]-T and the sequences downloaded were inspected visually. A list with the bacterial reference organism names containing CDF poly-his, P_IB4_-ATPase or both was generated and used to create a Venn diagram.

### Metal sensitivity determination

Metal sensitivity was assessed by inhibition halo experiments and the agar dilution method. In the first case aliquots of 5 ml molten LB-soft agar media (agar 0.6%) containing approximately 5.10^7^ cfu/ml (OD_600 nm_ = 0.05) were laid on LB-agar plates. After solidification four dried filter paper discs (7-mm diameter) previously embedded with 10 μl of different metal solutions (1 M, 0.5, 0.2, and 0.1 M as chloride salts except for FeSO_4_) were placed on top of the agar. Sensitivity was determined as zones of clearing surrounding each disc and scored after 18 h incubation at 37°C. For the agar dilution method LB liquid cultures were grown till OD_600 nm_ of 1.0 from an overnight culture and 10 μl of serial dilution were spotted on LB-agar plates supplemented with the desired metal concentration as indicated in the figure.

### Pyoverdine detection

LB soft-agar plates with *P. aeruginosa* strains grown for 16 h from OD_600 nm_ = 0.005 were exposed to white light (300–700 nm) and UV light (<300 nm; UVP, CA). Images were obtained with a digital camera and the settings controlled by an image acquisition software (Doc-It, UVP). ROIs were selected and analyzed with ImageJ. ROIs were defined as the surrounding area of the Zn-loaded filter paper disc where a fluorescent signal is detected and there is no inhibition of growth. Optical density signals were obtained from mean gray value intensities of the fluorescent signal area, normalized by those obtained from white light (300–700 nm) exposed plates. The value from WT strain was subtracted in all cases. Strains with the *pvdA* gene disrupted showed no fluorescent signal after 2 days of incubation in cetrimide-agar plates. This was interpreted as incapability to produce the siderophore pyoverdine in the strains.

### Metal accumulation

Fifty milliliters of liquid LB cultures in late exponential phase were supplemented with 0.1 mM CoCl_2_, ZnCl_2_ or FeSO_4_ and incubated for 2 h. End point inhibitory growth curves showed these concentrations to be in the sub-lethal range. After this incubation, OD_600 nm_ was determined, cells harvested, and washed with 0.9% NaCl. Pellets were digested with 0.5 ml of HNO_3_ (trace metal grade) for 1 h at 80°C and then overnight at 20°C. Digestions were stopped by the addition of 0.1 ml of 30% H_2_O_2_ and dilution to 10 ml with water. Metal contents in samples were measured by ICP–MS.

### Membrane permeability assays

The LB liquid cultures were inoculated at OD_600 nm_ of 0.1 from overnight cultures. When an OD_600 nm_ value of 0.6 was reached, cultures were washed and resuspended in 5 mM HEPES-Na, pH 7.2. Three fractions were used for different treatments. One was supplemented with 1 mg/ml lysozyme, another was supplemented with 1 mg/ml lysozyme plus 1 mM EDTA and the rest was left unamended. Cells were counted at 0, 5, 10, and 15 min after addition of the reagents. Survival rate was estimated as the ratio of number of cells in the presence and the absence of any treatment.

### Antibiotic susceptibility determination

Antibiotic susceptibility was assessed by the agar dilution method in Müller–Hinton medium. For imipenem minimal inhibitory concentration (MIC) was corroborated by the Epsilon test (Biomerieux Diagnostics) in Müller–Hinton medium and according to manufacturer instructions.

### Outer membrane proteins purification and MS-MS analysis

Cells were harvested from 200 ml LB cultures during the late exponential growth phase via centrifugation (6,000 × g, 10 min, 4°C) and washed twice with 10 ml of 10 mM Tris-HCl (pH 7.5). The cell suspensions were then sonicated using 2 cycles of 20 s bursts at 80–120 W on ice. Any intact cells were removed via 10 min of centrifugation at 6,000 × g at 4°C. The supernatants were centrifuged for 30 min at 100,000 × g at 4°C. After washing the pellets with 10 mM Tris-Cl pH 7.5, they were resuspended in 30 ml of 10 mM Tris-Cl pH 7.5, Triton X-100 was added to a final concentration of 2% and incubated for 10 min at room temperature. The Triton X-100- insoluble fraction was then separated via high-speed centrifugation (100,000 × g, 1 h). The pellets were washed in 30 ml 10 mM Tris-Cl pH 7.5 once and finally resuspended in 200 μl 10 mM Tris-Cl pH 7.5. Protein was measured in accordance to Bradford (1976) and resolved in SDS-PAGE 8%. Gels were stained with Coomassie Brillant Blue and bands analyzed visually. A 50 kDa band showing higher intensity in the mutant strains was excised for tryptic digestion and MS/MS identification. MS/MS determinations were performed at the Institut Pasteur–Analytical Biochemistry and Proteomics Unit- Montevideo, Uruguay.

### Hydrogen peroxide sensitivity test

The LB liquid cultures were inoculated at OD_600 nm_ of 0.1 from overnight cultures. When an OD_600 nm_ value of 0.6 was reached, cultures were split, a half was supplemented with 30 mM H_2_O_2_ and the other left unamended. Cells were counted at 0, 15, 30, and 45 min after adding H_2_O_2_. Survival rate was estimated as the ratio of number of cells in the presence and the absence H_2_O_2_.

### Infectivity assay

*Arabidopsis thaliana* ecotype Columbia was infected with *P. aeruginosa* as described by Baldini et al. (2014). Briefly, 3-week-old plants were inoculated by leaf infiltration with approximately 2 × 10^6^ colony-forming units (cfu) per milliliter (OD_600 nm_ 0.002). Two leaf discs were taken from each infected plant at 0, 3, and 4 days post infection (d.p.i.) and homogenized in 10 mM MgSO4. Serial dilutions of this homogenate were plated in selected media and cfu were counted after 16 h growth at 37°C. Four plants were infected per each bacterial strain.

## Results

### Bioinformatics analyses

The transport mechanism of CDF transporters requires TM binding at the intracellular exposed site A for subsequent export. Considering that optimal coordination at the transport site depends on lateral amino acids side chain the specificity for metal transport can be predicted a priori *in silico*. *P. aeruginosa* PAO1 CDF transporter sequences were analyzed by multiple sequence alignment including the archetypical Zn/Fe/Cu CDF transporters, YiiP from *E. coli* and CzcD from *Bacillus subtilis* (Grass et al., 2005; Moore et al., 2005). The alignment indicates conservative substitutions at the transport metal binding site A in the three *P. aeruginosa* paralogous genes with lateral amino acids chains having O (Asp and Glu) or N (His) ligands (Figure 1A, blue dots). PA3963/YiiP has the highest similarity with the archetypical member *E. coli* YiiP showing strict identity for residues forming sites A, B, and C (Figure 1A, blue, green, and cyan dots). Residues in sites A, B, and C of PA0397/CzcD are similar to residues found in the previously characterized *B. subtilis* CDF member CzcD with a conservative substitution at TMS 5 (His153-Glu153). In *B. subtilis*, CzcD confers resistance to Zn^2+^, Co^2+^, Ni^2+^, and Cu^+/2+^ (Moore et al., 2005). Another characterized ortholog, *zitA*, was included in the analyses as there is evidence pointing to a role for this gene in Zn^2+^ homeostasis in *Mycobacterium smegmatis* (Grover and Sharma, 2006; Raimunda et al., 2012). *P. aeruginosa* CzcD shares strict identity at the metal transport site with *zitA* (Figure 1A) and these clustered together (Figure 1B) indicating a likely participation in Zn^2+^ transport. The presence of a cytosolic poly-histidine stretch between TMS 4 and TMS 5 in PA1297/AitP suggests that this CDF is involved in Co^2+^ fluxes as similar low complexity histidine-rich sequences are found in Co^2+^ transporting members (Montanini et al., 2007; Podar et al., 2012). Interestingly, *P. aeruginosa* PAO1 genome lacks a P_IB4_-ATPase. Members of this sub-family have been characterized to participate in Co^2+^/Fe^2+^ transport in bacterial pathogens (Patel et al., 2016). This led us to inquire whether the presence of a CDF showing a poly-his stretch between TMS 4 and TMS 5 conditions negatively the presence of a P_IB4_-ATPase in other bacteria. The analysis of 4,430 bacterial reference genomes indicated that 553 contained at least one of the two proteins (Supplementary Table 1), i.e., a CDF with a poly-his stretch or an Fe^2+^/Co^2+^-ATPase and that a low percentage (6.3%) of these genomes contained both a CDF with a poly-his stretch and an Fe^2+^/Co^2+^-ATPase (Figure 1C). Taken together, these analyses suggested that AitP could play a role in Fe^2+^ export.

### Metal sensitivity screening of *P. aeruginosa* CDFs mutants

**Figure 2:**
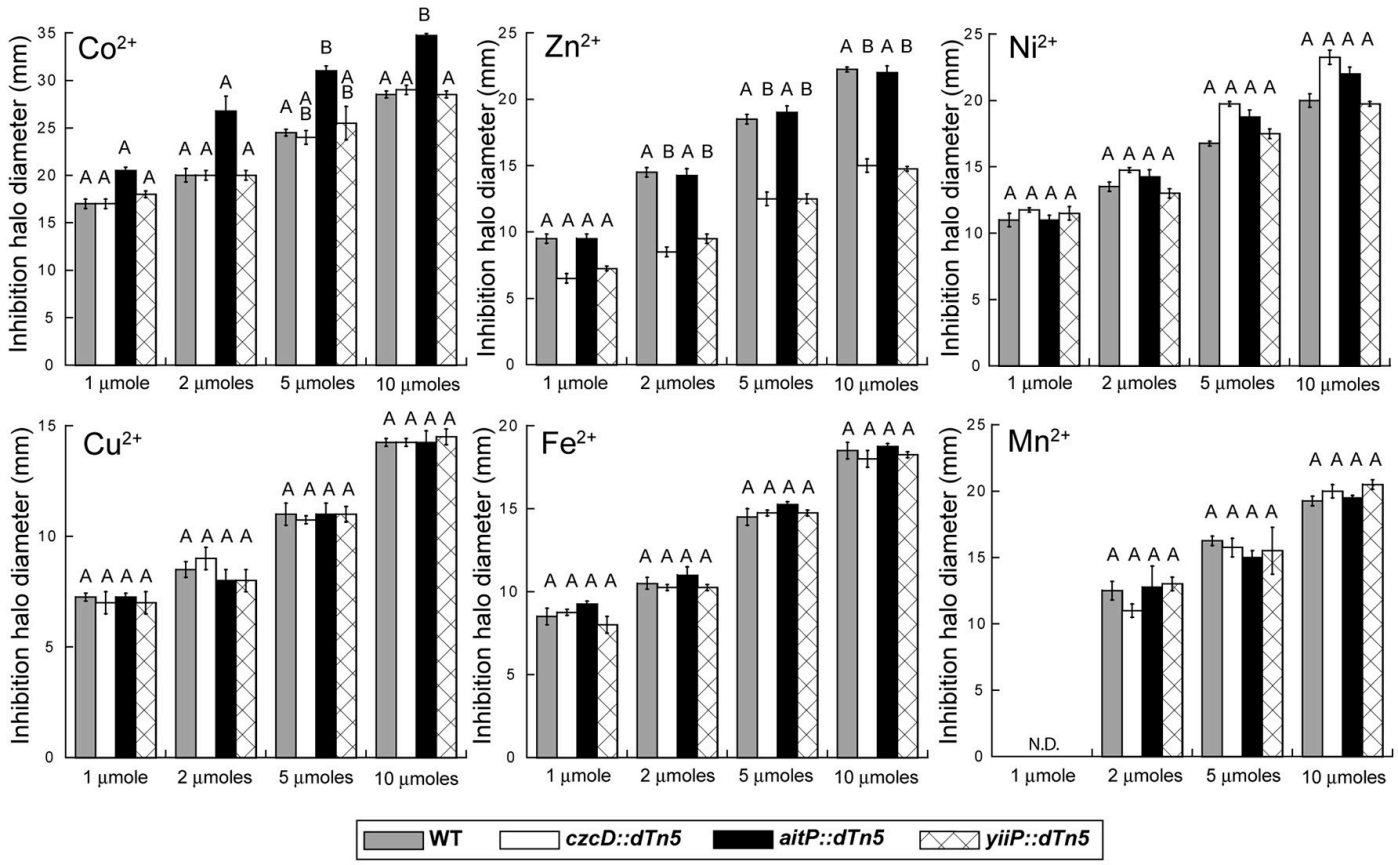
**Screening of *P. aeruginosa* CDF transporters roles in transition metal homeostasis and transport**. Metal toxicity was evaluated by inhibition halo experiments in strains PAO1 (gray bars), *czcD::dTn5* (empty bars), *aitP::dTn5* (black bars) and *yiiP::dTn5* (cross-hatched bar). 7-mm filter paper discs loaded with several amounts (x-axis) of metal (upper left in panels) were dried and then laid on top of a soft-agar inoculated with each strain (OD_600 nm_ = 0.05). Plates were incubated 16 h at 37°C and inhibition halo diameters measured. N.D. = not detected. Bars indicate mean ± SE of three independent experiments. Different letters designate significantly different means as informed by a Bonferroni *post hoc* (*p* < 0.05) test of an ANOVA.

We initially screened the TM sensitivity of the loss-of-function mutants for CzcD, AitP and YiiP vs. WT, by inhibition halo experiments. An increased Co^2+^ sensitivity was observed for the strain *aitP::dTn5* (Figure 2). A subtle Fe^2+^ sensitivity was detected in this strain also. Unexpectedly, the strains *czcD::dTn5* and *yiiP::dTn5* showed Zn^2+^ resistance while no changes were detected with other metals. Ni^2+^ sensitivity was apparent for strains *czcD::dTn5* and *aitP::dTn5* only at higher metal concentrations. This and the fact that total Ni^2+^ concentrations in biological systems are much lower than Fe^2+^ and Zn^2+^ precluded us to test further the participation of CzcD and AitP in Ni^2+^ homeostasis.

### AitP is involved in Fe^2+^ and Co^2+^ homeostasis

**Figure 3:**
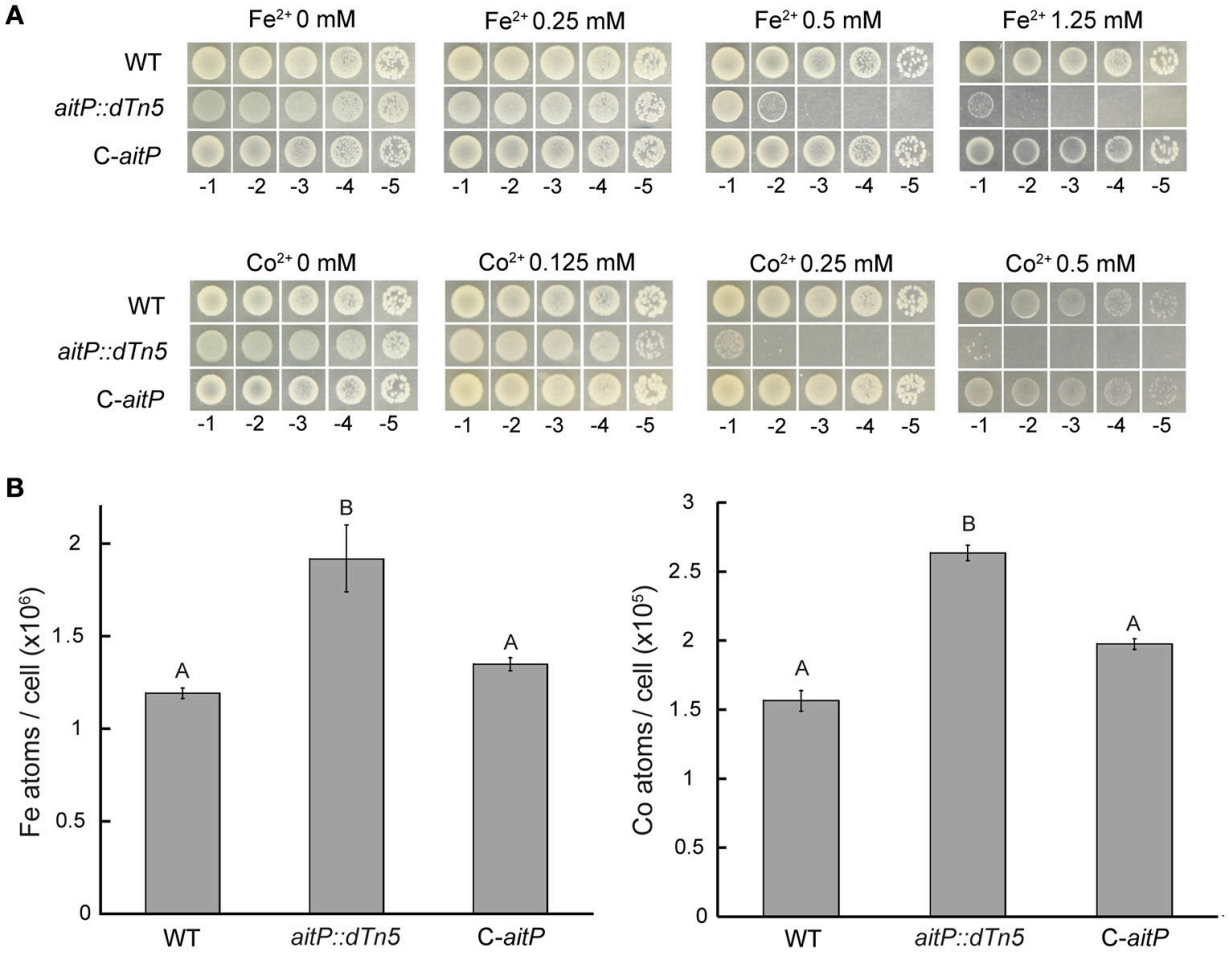
**AitP is involved in Fe^2+^ and Co^2+^ homeostasis**. Serial dilutions (10 μl) of *P. aeruginosa* PAO1 (WT), *aitP::dTn5* and complemented C-*aitP* cells initially at OD_600 nm_ = 1.0 were spotted on LB-agar supplemented with the indicated Fe^2+^ (as ${\text{SO}}_{4}^{2-}$) or Co^2+^ (as Cl^−^) concentration and incubated for 16 h at 37°C **(A)**. Iron and cobalt content was measured in WT, *aitP::dTn5* and C-*aitP* after incubation with 0.1 mM FeSO_4_ or CoCl_2_ for 2 h in LB medium during early exponential phase growth. Metal contents are shown as atoms/cell **(B)**. Data are the mean ± SE of three independent experiments. Different letters designate significantly different means as informed by a Bonferroni *post hoc* (*p* < 0.05) test of an ANOVA.

Inhibition halo experiments in soft LB-agar showed that the insertional mutant of *aitP* was sensitive to Co^2+^ and probably to Fe^2+^ compared to the WT strain. These phenotypes were confirmed by the dilution agar method (Figure 3A) and reverted in the complemented strain C-*aitP* discarding polar effects. Growth inhibition could be the consequence of intracellular Fe^2+^ and Co^2+^ accumulation due to the lack of transport in this loss-of-function mutant. To test this intracellular Fe^2+^ and Co^2+^ content was measured in cells incubated at the exponential phase in presence of a sub-lethal concentration of 100 μM Fe^2+^ or Co^2+^ for 2 h. Compared to WT, the strain *aitP::dTn5* showed a 60–70% increase in Fe^2+^ and Co^2+^ accumulation (Figure 3B). This suggests a direct participation of AitP in these ions homeostasis as an Fe^2+^/Co^2+^ exporter.

### CzcD and YiiP export Zn^2+^

**Figure 4:**
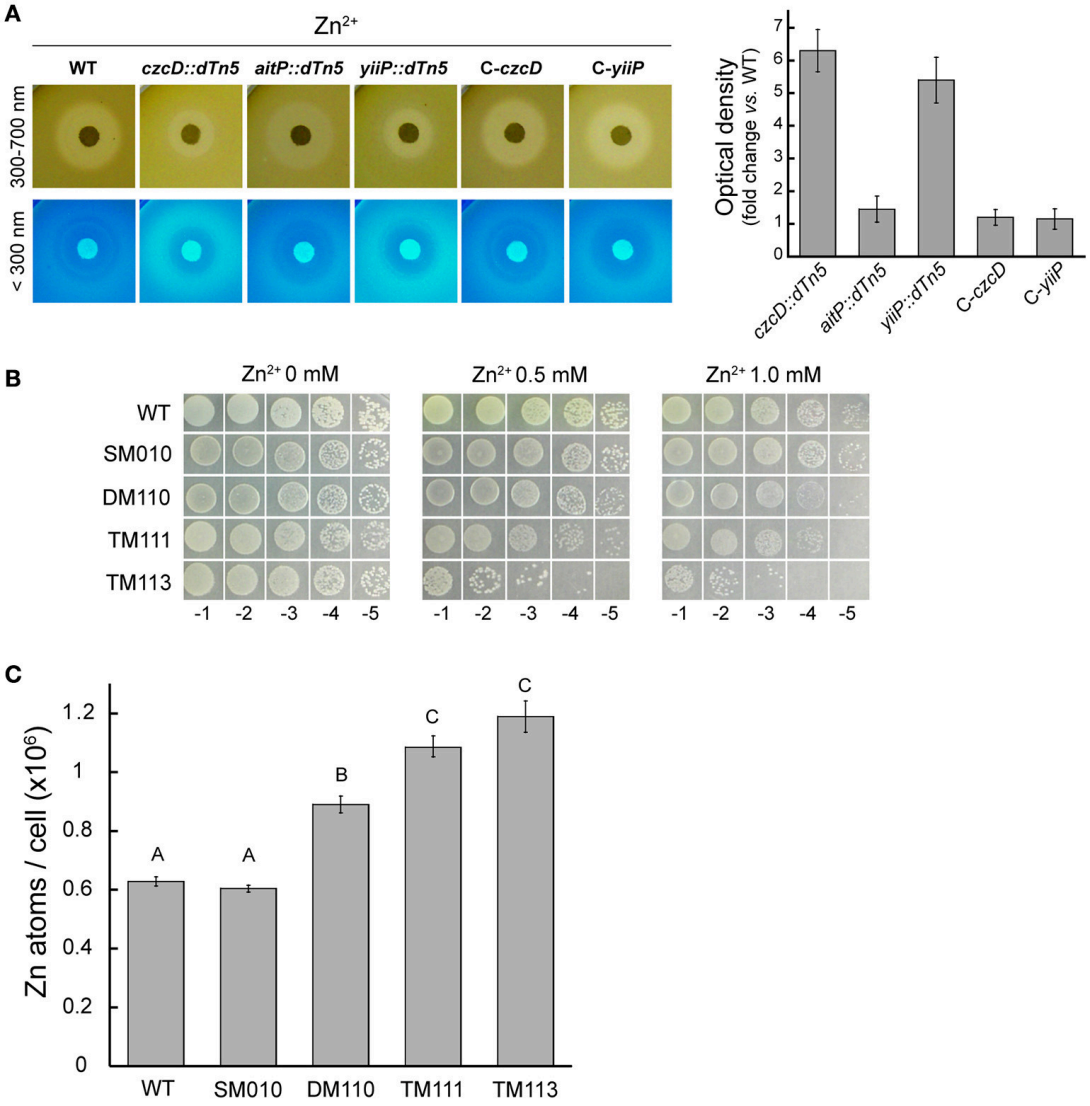
**CzcD and YiiP play a role in Zn^2+^ transport**. Differential Zn^2+^-dependent pyoverdine production was estimated by relating optical densities of images acquired using UV light (<300 nm) to those acquired using white light (300–700 nm) as described in detail in the Materials and Methods section. Strains were grown for 16 h at 37°C in soft-agar plates containing Zn^2+^-loaded filter discs (5 μmoles). Bars indicate fold increase in fluorescence vs. WT. Note the increased resistance to Zn^2+^ and the increased fluorescent signal in strains *czcD::dTn5* and *yiiP::dTn5* vs. WT **(A)**. Serial dilutions (10 μl) of *P. aeruginosa* strains initially at OD_600 nm_ = 1.0 were spotted on LB-agar supplemented with the indicated Zn^2+^ concentration (as Cl^−^) and incubated for 16 h at 37°C **(B)**. Zinc content was determined in *P. aeruginosa* strains after incubation with 0.1 mM ZnCl_2_ in LB medium for 2 h during early exponential phase growth. Metal contents are shown as atoms/cell **(C)**. WT (PAO1), SM010 (*pvdA* mutant), DM110 (*zntA* and *pvdA* mutant), TM111 (*zntA, pvdA*, and *czcD* mutant) and TM113 (*zntA, pvdA*, and *yiiP* mutant). Data are the mean ± SE of three independent experiments. Different letters designate significantly different means as informed by a Bonferroni *post hoc* (*p* < 0.05) test of an ANOVA.

Inhibition halo experiments showed that *czcD::dTn5* and *yiiP::dTn5* are more resistant to Zn^2+^ (Figures 2, 4A). When the Zn^2+^ disc-containing plates were observed under UV light a fluorescent halo was detected surrounding the inhibition zone (Figure 4A). This halo had higher fluorescent intensities in the cases of these strains (Figure 4A). Although there was a variation in the fluorescence signal of the strains tested with Ni^2+^, Cu^2+^, Fe^2+^, and Mn^2+^ (not shown), only Zn^2+^ produced a consistently defined fluorescent halo throughout the experiments. It is well known that *P. aeruginosa* secretes the green fluorescent molecule, pyoverdine, in order to acquire Fe^2+^ (Vasil and Ochsner, 1999). Intracellular iron levels were similar to the WT in both strains grown in presence of FeSO_4_ (not shown), discarding a putative role in Fe^2+^ import. However, it has previously been described that the presence of elevated Zn^2+^ in the media induces pyoverdine production in *P. aeruginosa* (Hofte et al., 1993; Rossbach et al., 2000). Mutation of *pvdA* in the background mutants suppresses the Zn^2+^-dependent response supporting that pyoverdine and no other fluorophore yields the fluorescent halo (Supplementary Figure 1). Considering this and the fact that pyoverdine is able to chelate divalent TM other than Fe^2+^ with high affinities (Schalk and Guillon, 2013), the Zn^2+^ resistant phenotypes of *czcD::dTn5* and *yiiP::dTn5* may be explained by the overproduction of the chelator in response to a transient cytosolic Zn^2+^ increase. Although we observed a subtle increased in Zn^2+^ sensitivity when *czcD* or *yiiP* where disrupted in a non-pyoverdine producer strain, the existence of the gene *zntA* codiyng for a Zn^2+^-ATPase in *P. aeruginosa* genome precluded us to asses phenotypically a direct role in Zn^2+^ transport of CzcD and YiiP. Thus, we created the triple mutants lacking ZntA, PvdA and each of the CDF, CzcD, or YiiP (Table 1, TM111 and TM113), and compared the Zn^2+^ sensitivity of these vs. the double mutant lacking ZntA and PvdA (Table 1, DM110). We observed an increased sensitivity in both cases (Figure 4B) which correlated with an increased intracellular accumulation of Zn^2+^ (Figure 4C).

### CzcD and YiiP confers resistance to a wide range of antibiotics

The increased sensitivity to Zn^2+^ observed in the triple mutant strains suggests that the roles of both CDF in this bacterium are linked to Zn^2+^ efflux. However, the presence of a Zn^2+^ transporting P_IB_-ATPase in the *P. aeruginosa* genome argues against the participation of CzcD and YiiP in Zn^2+^ tolerance. We noticed that of the three Tc^r^ transposon mutant strains, *yiiP::dTn5* and *czcD::dTn5* had a MIC tetracycline concentration decreased by half. Evaluation of MICs for several antibiotics showed a 4–8-fold increased sensitivity to imipenem and ciprofloxacin and a 2-fold increased sensitivity to chloramphenicol and gentamicin for *yiiP::dTn5* and *czcD::dTn5* (Table 2). Interestingly, the MIC-values remained unchanged after *pvdA* was disrupted in these mutants discarding a possible participation of pyoverdine in the mechanism leading to increased antibiotic sensitivity.

**Table 2:** **Antibiotic susceptibility of *P. aeruginosa* wild-type and CDF-mutant strains**.

**Strain**	**MIC (μg/ml)**			
	**^a^*CP***	**GM**	**CIP**	**IMI**
PAO1	256	1	1	2
*aitP::dTn5*	256	1	1	2
*czcD::dTn5*	128	0.5	0.125	0.5
*yiiP::dTn5*	128	0.5	0.125	0.5
DM011^b^	128	0.5	0.125	0.5
DM013	128	0.5	0.125	0.5
C-*czcD*	256	N.D.^c^	1	2
C-*yiiP*	256	N.D.	1	2

a *CP, chloramphenicol; GM, gentamicin; CIP, ciprofloxacin; IMI, imipenem*.

b *In this nomenclature the first digit correspond to zntA gene, the second to pvdA gene and the third to CDF genes. Zero means no mutation of the gene while 1 indicates mutation of the gene. For CDFs, 1 means czcD mutation, and 3 means yiiP mutation*.

c *N.D., not determined*.

### Outer membrane stability depends on CzcD and YiiP

**Figure 5:**
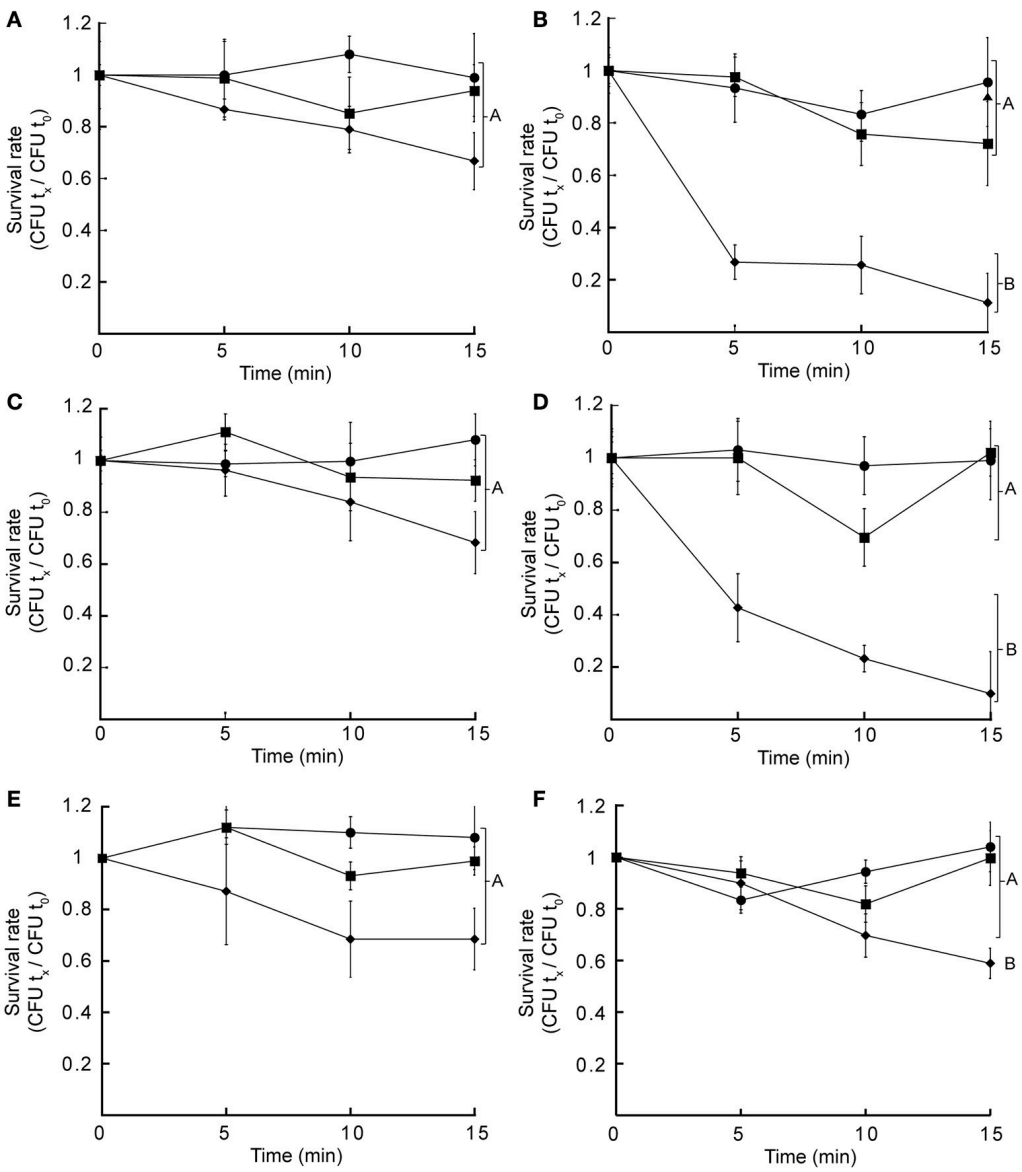
**Insertional mutant strains of CzcD and YiiP show altered permeability of the outer membrane**. Wild type **(A)**, *czcD::dTn5* **(B)**, *aitP::dTn5* **(C)**, *yiiP::dTn5* **(D)**, C-*czcD* **(E)**, or C-*yiiP* **(F)** at mid-exponential phase were incubated in a low osmolarity buffer alone (•), supplemented with 1 mg/ml lysozyme (■) or with 1 mg/ml lysozyme plus 1 mM EDTA (♦). After the indicated time cells were estimated and normalized to cell number at time = 0 (CFU t_x_/CFU t_0_). Data are the mean ± SE of three independent experiments. Different letters designate significantly different means as informed by a Bonferroni *post hoc* (*p* < 0.05) test of a two-way ANOVA.

Hydrophobic and hydrophilic antibiotics can permeate the outer membrane via lipid-mediated pathway or via diffusion porins, respectively (Delcour, 2009). Thus, the observed sensitivity toward several hydrophobic antibiotics in *czcD::dTn5* and *yiiP::dTn5* might reflect alterations in the outer membrane as this is constituted mostly by lipopolysaccharides (LPS). To test this we analyzed the survival rate of grown cells incubated in presence of lysozyme alone or lysozyme plus EDTA in a low osmolarity buffer. This divalent cation chelator is hypothesized to alter the LPS distribution in the outer membrane allowing lysozyme to disrupt the cell wall with consequent cell lysis. A two-way ANOVA analysis indicated that in all cases the time variable may affect cell survival (two-way ANOVA, *p* < 0.05). Furthermore, the same analysis showed that the treatment variable significantly changed the cell growth rates of *czcD::dTn5, yiiP::dTn5* (two-way ANOVA, *p* < 0.0001) and C-*yiiP* strains (two-way ANOVA, *p* < 0.002). Multiple comparisons at each time points by corrected *post hoc* test showed that significant differences in the survival rate of these strains were only attained when cells were incubated with lysozyme plus EDTA (Figure 5). The same analysis pointed to no significant differences between samples of the same treatment for WT, *aitP::dTn5* and C-*czcD*. The complemented strains C-*czcD* and C-*yiiP* become resistant similar to the control treatment levels except in the C-*yiiP* strain where a partial restoration of the phenotype was observed (Figures 5E,F). These results indicate that the outer membrane permeability toward hydrophobic compounds and large macromolecules might be affected in *czcD::dTn5* and *yiiP::dTn5*.

### CzcD or YiiP mutation results in altered OprN and OprD expression

**Figure 6:**
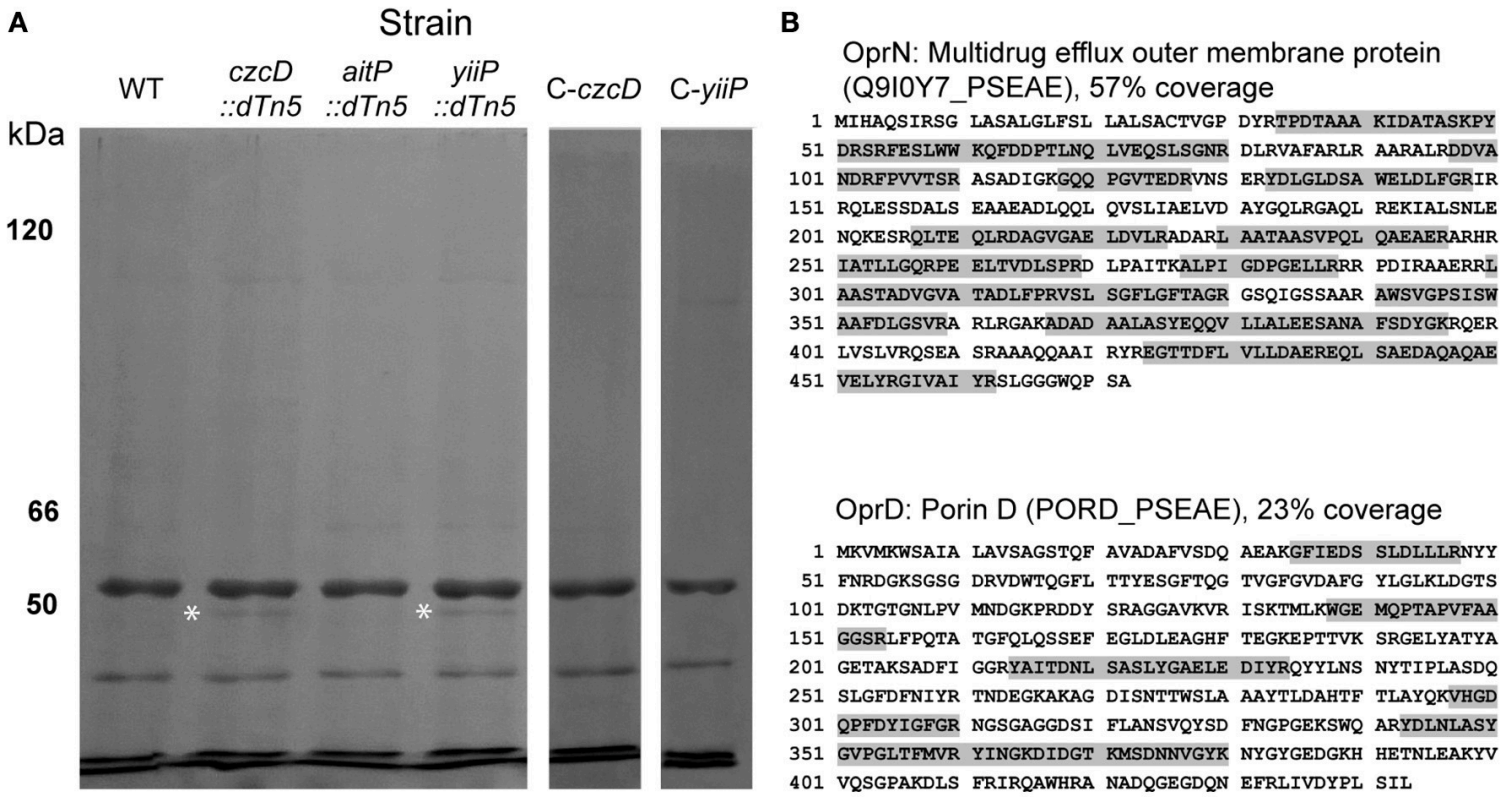
**Insertional mutation of CzcD and YiiP induces overexpression of outer membrane proteins OprN and OprD**. SDS-PAGE analysis of outer membrane protein fractions from *P. aeruginosa* strains. Proteins were resolved in an 8% gel and stained with Coomassie Brillant Blue. White asterisks indicate the position of the bands observed only in fractions of *czcD::dTn5* and *yiiP::dTn5* **(A)**. The bands were excised and identified by MS/MS. Detected OprN and OprD peptides (gray highlighted letters) and maximal sequence coverage are shown **(B)**.

The increased sensitivity toward more hydrophobic antibiotics such as chloramphenicol, gentamicin and ciprofloxacin can be explained considering that the outer membrane permeability is compromised in the CzcD and YiiP mutant stains (Figure 5). However, small hydrophilic compounds, like imipenem, require outer membrane porins for cell entry (Eren et al., 2013). In *P. aeruginosa*, the OprD porin enables the entry of positively charged aminoacids and imipenem (Trias and Nikaido, 1990). OprD expression is negatively regulated in response to incremental Zn^2+^ levels in the growth media through the two component system CzcRS (Perron et al., 2004; Dieppois et al., 2012). Also the global regulator factor MvaT negatively affects *oprD* expression (Westfall et al., 2006; Lister et al., 2009). In order to evaluate changes in outer membrane protein expression leading to increased antibiotic sensitivity, we analyzed the protein profile of this fraction in all strains by SDS-PAGE. A 50–55 KDa band was differentially expressed in both *czcD::dTn5* and *yiiP::dTn5* (Figure 6A). The band was analyzed by MS/MS and the peptides identified matched with OprN and OprD (Figure 6B). These proteins could not be detected in *aitP::dTn5* and WT samples. In *P. aeruginosa*, together with MexE and MexF, OprN forms an RND (resistance-nodulation-division) efflux pump characterized to participate in ciprofloxacin and the P*seudomonas* quinolone signal (PQS, 2-heptyl-3-hydroxy-4-quinolone) efflux. The increased expression of OprD correlates with the imipenem sensitivity shown by *czcD::dTn5* and *yiiP::dTn5*. Although the OprN overexpression does not correlate with the ciprofloxacin sensitivity observed in these strains (Table 2), it may be a consequence of outer membrane permeability changes leading to intracellular accumulation of hydrophobic PQS molecules, the natural substrate of the MexEF-OprN RND efflux system.

### *P. aeruginosa* CDFs participates in virulence

**Figure 7:**
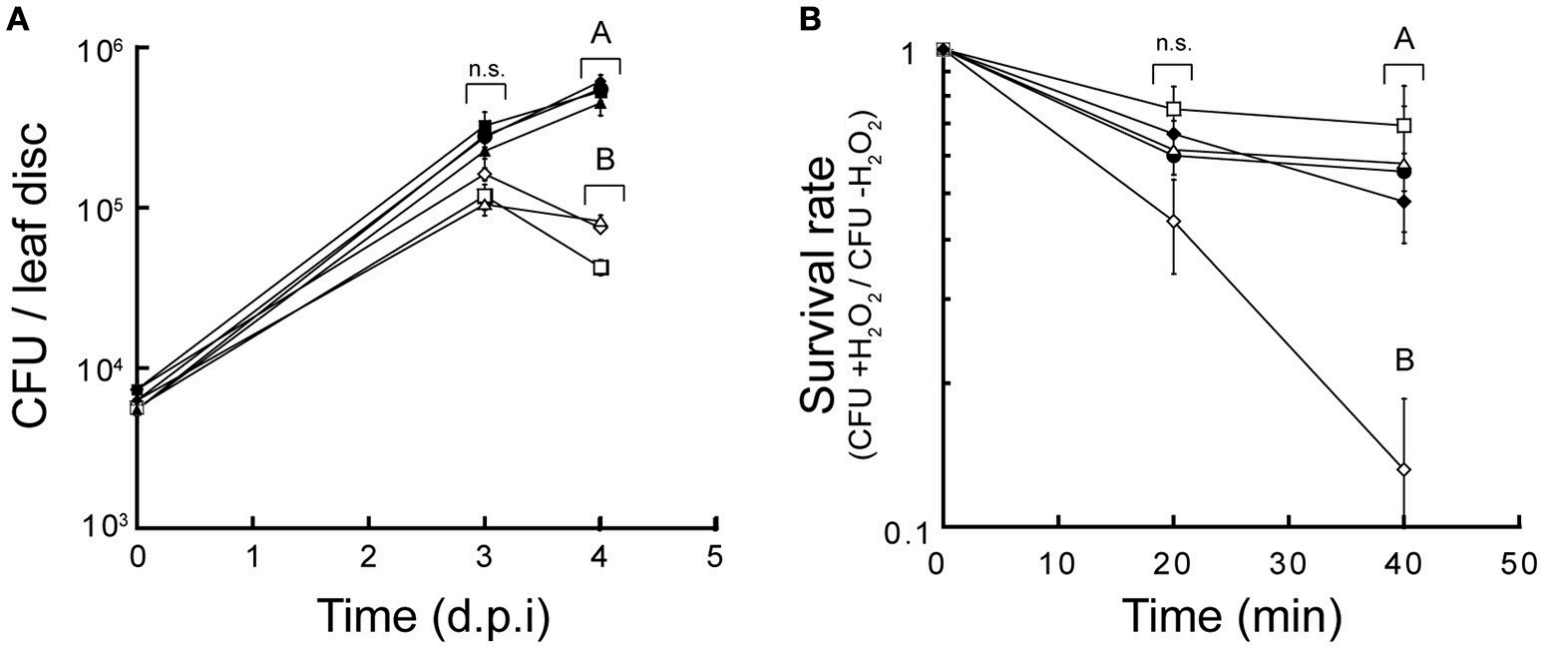
***P. aeruginosa* CDF transporters are required for virulence playing different roles**. Growth rate of *P. aeruginosa* PAO1 (•), *czcD::dTn5* (□), *aitP::dTn5* (♢), and *yiiP::dTn5* (Δ) in *A. thaliana* leaves. Complemented strains of the CDF mutants are shown (C-*czcD*, ■; C-*aitP*, ♦; C-*yiiP*, ▲) **(A)**. Cell survival of *P. aeruginosa* PAO1 (•), *czcD::dTn5* (□), *aitP::dTn5* (♢), *yiiP::dTn5* (Δ) and *aitP* complemented (C-*aitP*, ♦) after treatment with 30 mM H_2_O_2_ in LB medium for various lengths of time. Cell viability is shown as the ratio of CFU of treated/untreated cultures **(B)**. Data are the mean ± SE of three independent experiments. Different letters designate significantly different means as informed by a Bonferroni *post hoc* (*p* < 0.05) test of an ANOVA. n.s, not statistically significant.

It was previously shown that members of the CDF family responsible for Mn^2+^ homeostasis play a crucial role in bacterial virulence (Rosch et al., 2009; Raimunda and Elso-Berberian, 2014). To evaluate possible roles in virulence of CzcD, AitP, and YiiP a simple assay that takes advantage of *P. aeruginosa* capability to infect plants (Baldini et al., 2014) was used. Figure 7A shows that the three *P. aeruginosa* CDFs were required for host infection. As oxidative burst with generation of H_2_O_2_ and free radicals is part of the defense mechanism in plants (Wojtaszek, 1997) we tested *in vitro* the resistant to H_2_O_2_. Only *aitP::dTn5* showed decreased survival in presence of the stressor (Figure 7B). This indicates that their roles are achieved by different mechanisms probably related to the transport specificity. Complementation with the wild-type genes conferred the strains normal virulent phenotypes and normal levels of resistance to H_2_O_2_.

## Discussion

Previous studies have shown that bacterial CDF transporters are involved in TM tolerance establishing their participation in TM export to avoid cellular toxicity through intracellular metal accumulation (Grass et al., 2005; Grover and Sharma, 2006; Rosch et al., 2009). Although their roles during bacterial infection was also described (Rosch et al., 2009), in *P. aeruginosa* these were not identify as relevant factors for wound infection or for intrinsic antibiotic resistance (Turner et al., 2014; Murray et al., 2015). However, a study of the genes required to colonize the murine gastrointestinal tract showed that a CzcD homolog plays an important role in *P. aeruginosa* PA14 during this process (Skurnik et al., 2013). Independent of the methodological aspects leading to these differences, our results show that the three members of CDF family in *P. aeruginosa* PAO1 are required for proper infection fitness in the *A. thaliana* infection model. This has led us to evaluate a possible correlation between CDF specificity of transport and virulence. Another aim of this work was to test a possible functional redundancy among them or members of different TM transport protein families with similar specificity of transport, i.e., P_IB_-ATPases.

Protein sequence alignments of the *P. aeruginosa* CDF with previous archetypical characterized members, *B. subtilis* CzcD and *E. coli* YiiP, showed a high degree of conservation in amino acids at positions of the metal transport site. Positions of the cytosolic regulatory site C also showed a high degree of conservation except for the member AitP. A histidine-rich domain located between TMS 4 and TMS 5 in this member suggested a role in Co^2+^ transport (Montanini et al., 2007; Podar et al., 2012). As expected, the mutant strain lacking AitP showed increased sensitivity to Co^2+^ and Ni^2+^, although the last was observed only at higher Ni^2+^ concentrations. Supporting a role in Co^2+^ homeostasis this mutant accumulated more Co^2+^ vs. WT. Analysis of the *P. aeruginosa* genome indicates a lack of P_IB4_-ATPase. This sub-group of transporters has been characterized as Fe^2+^/Co^2+^-transporting ATPases (Guan et al., 2015; Patel et al., 2016) and to be required for virulence in pathogenic bacteria (Raimunda et al., 2014). The mutant lacking AitP showed increased sensitivity to Fe^2+^ and accumulated higher intracellular levels of this ion vs. WT. The nutritional immunity paradigm points to a lessen bioavailability of Fe^2+^ in the bacterial environment during infection (Hood and Skaar, 2012). A role for an Fe^2+^-transporting CDF emerges when considering Fe-S cluster Fe^2+^ released induced by the H_2_O_2_ generation in the activated macrophage with subsequent intracellular reactive oxygen radicals formation through Fenton reaction (Imlay, 2008). A sequence analysis of organisms having a P_IB4_-ATPases or a CDF poly-his stretches between TMS 4 and TMS 5 in their genome resulted in only 6% of the total organisms having both proteins (35/553). The fact that 78% of the reference genomes analyzed (553/4430) lacking a P_IB4_-ATPase and a CDF with a poly-his stretch raises the question on whether other transporters from these or other families participates in the Fe^2+^ efflux process.

Based on sequence similarity, the alignment shows a closer phylogenetic relationship between *B. subtilis* CzcD and *P. aeruginosa* CzcD and between *E. coli* YiiP and *P. aeruginosa* YiiP. We also included the only Zn^2+^-transporting CDF member present in *M. smegmatis* ZitA (Grover and Sharma, 2006; Raimunda et al., 2012). The same alignment shows that ZitA is phylogenetically related to CzcD. These results suggested that CzcD and YiiP are Zn^2+^ transporters. We demonstrated that CzcD and YiiP export intracellular Zn^2+^. The triple mutant strains lacking pyoverdine, the putative Zn^2+^-ATPase, ZntA, and CzcD or YiiP showed increased Zn^2+^ sensitivity and accumulated the ion at higher levels vs. the double mutant strains lacking pyoverdine and ZntA but expressing CzcD or YiiP. Considering the higher apparent Zn^2+^ accumulation of the triple mutants, vs. the double mutant, CzcD and YiiP would have access to different intracellular Zn^2+^ pools other than ZntA. The Zn^2+^-resistant phenotypes of the *czcD::dTn5* and *yiiP::dTn5* were unexpected and a parsimonious model explaining this would imply a Zn^2+^ importer function for both CDF members. However, the concomitant pyoverdine overproduction seen in these mutants in the presence of Zn^2+^ leads us to hypothesize a role in the efflux of this metal. It is known that pyoverdine synthesis genes are up-regulated in the presence of Zn^2+^ (Rossbach et al., 2000). Independent of the mechanism leading to pyoverdine overproduction, we hypothesize that Zn^2+^ toxicity is decreased in both mutants due to a lesser bioavailability of the metal in the media from the pyoverdine-Zn complex formation. Several conditions are required for this scenario to be possible. A transient rise in the cytosolic Zn^2+^ concentration would be necessary to induce pyoverdine synthesis in these strains. Moreover, although normal intracellular Zn^2+^ pools could be restored eventually, the binding of pyoverdine-Zn complexes to FpvA could amplify the signal in a positive feedback for pyoverdine synthesis (Braud et al., 2009).

Considering the Zn^2+^ transport specificity of CzcD and YiiP and the phenotypes observed in *czcD::dTn5* and *yiiP::dTn5* indicating antibiotic susceptibility, outer membrane permeability changes and overexpression of outer membrane proteins, two likely scenarios are hypothesized. First, CzcD and YiiP may participate in Zn^2+^ efflux to fine tune the TM intracellular homeostasis. Changes in Zn^2+^ levels could trigger pleiotropic effects, leading to outer membrane defects and instability. Alternatively, Zn^2+^ transport could be coupled to periplasmic Zn^2+^ sensing via two component systems. This supposes a protein-protein interaction and a Zn^2+^ transfer reaction from the CDF to the sensing component at the periplasmic side of the inner membrane. In *P. aeruginosa* the two component systems CzcRS and ColRS have been described as participating in Zn^2+^ sensing leading to Zn^2+^ and antibiotic resistance and LPS modifications, respectively (Perron et al., 2004; Nowicki et al., 2015). Transcription of *oprD* is regulated in the periplasm via CzcRS. When Zn^2+^ efflux is downregulated, as in the *czcD::dTn5* and *yiiP::dTn5*, an increase in *oprD* transcription is expected. A direct positive effect on *oprD* expression by extracellular pyoverdine-Zn^2+^ formation is discarded as the non-pyoverdine producers showed the same sensitivity toward imipenem and the others antibiotics (Table 2). The Zn^2+^ content in our media (5 μM by ICP-MS) is sufficient to suppress *orpD* expression in the WT (Perron et al., 2004). Although purely speculative, this would not be the case in the *czcD::dTn5* and *yiiP::dTn5* strains, where OprD is overexpressed relative to the WT and *aitP::dTn5* strains.

How can the twin phenotypes shown by the single mutant strains *czcD::dTn5* and *yiiP::dTn5* be explained? Since the CDF functional unit is a dimer, we hypothesize that CzcD and YiiP form a functional heterodimer. Residues at positions forming the charge interlock at the cytosol-membrane interface in *E. coli* YiiP, i.e., Lys77 and Asp207, are well conserved in CzcD and YiiP and thus the heterodimer formation is plausible (Kolaj-Robin et al., 2015). Recent studies proposed that in the magnetotactic bacteria *Magnetospirillum gryphiswaldense* two CDF genes, *mamB* and *mamM*, interact to form a heterodimer functional unit (Uebe et al., 2011).

In conclusion, this study provides evidence for new roles of CDF transporters required during bacterial infection. More specifically, our results show that Zn^2+^ efflux mediated by YiiP and CzcD is required in *P. aeruginosa* for proper outer membrane permeability. A third CDF member, AitP, is required for Fe^2+^/Co^2+^ homeostasis.

## Author contributions

Conceived and designed the experiments: DR; Performed the experiments: AS, DR; Analyzed the data: AS, DR; Contributed reagents/samples/analysis/tools: DR. Wrote or edited the manuscript: AS, DR; All authors read and approved the final manuscript.

## Funding

This work was supported by the Agencia Nacional de Promoción Científica y Tecnológica (PICT2013–2258 and PICT2015–2897 to DR). DR is a Consejo Nacional de Investigaciones Científicas y Tecnológicas (CONICET) investigator.

### Conflict of interest statement

The authors declare that the research was conducted in the absence of any commercial or financial relationships that could be construed as a potential conflict of interest. The reviewer MLV and handling Editor declared their shared affiliation and the handling Editor states that the process nevertheless met the standards of a fair and objective review.

## Abbreviations

TM: transition metal
CDF: cation diffusion facilitator
TMS: transmembrane segment
CTD: C-terminal domain
ICP-MS: inductively coupled plasma mass spectrometry.
