# Supplementary material for: Defining the Roles of the Cation Diffusion Facilitators in Fe2+/Zn2+ Homeostasis and Establishment of Their Participation in Virulence in Pseudomonas aeruginosa
Source: Front Cell Infect Microbiol. 2017 Mar 20;7:84. doi: 10.3389/fcimb.2017.00084 (PMC5357649; doi:10.3389/fcimb.2017.00084)
Supplement: Supplementary file 1 [file Table1.docx]

Supplementary Table 1. Alphabetical list of reference genome-sequenced bacterial organisms analyzed for the presence of Fe^2+^/Co^2+^-transporting PfeT-like ATPases (left column) and AitP-like CDF transporters (right column). In bold and pink background are highlighted the organisms with genomes having a P_IB4_-ATPases and a CDF-poly his.

| **P_IB4_-ATPase (PfeT-like)** | **CDF-poly his (AitP-like)** |
| --- | --- |
| *Acetobacter tropicalis* | *Acaryochloris marina* |
| *Achromobacter piechaudii ATCC 43553* | *Acetobacteraceae bacterium AT-5844* |
| *Achromobacter sp. LC458* | *Acidiphilium multivorum* |
| ***Acidovorax delafieldii*** | *Acidocella facilis* |
| ***Acidovorax ebreus*** | ***Acidovorax delafieldii*** |
| *Acidovorax sp. CCH12-A4* | ***Acidovorax ebreus*** |
| *Afipia broomeae ATCC 49717* | *Acinetobacter baumannii* |
| *Afipia felis* | *Acinetobacter bereziniae* |
| *Afipia sp. 1NLS2* | *Acinetobacter bohemicus ANC 3994* |
| ***Agrobacterium tumefaciens*** | *Acinetobacter bouvetii* |
| *Alcaligenes faecalis* | *Acinetobacter brisouii ANC 4119* |
| *Alicycliphilus denitrificans* | *Acinetobacter calcoaceticus/baumannii complex* |
| *Alishewanella agri* | *Acinetobacter gyllenbergii* |
| *alpha proteobacterium L41A* | *Acinetobacter indicus* |
| ***Altererythrobacter atlanticus*** | *Acinetobacter johnsonii* |
| *Altererythrobacter epoxidivorans* | *Acinetobacter junii CIP 64.5* |
| *Altererythrobacter namhicola* | *Acinetobacter lwoffii NCTC 5866 = CIP 64.10 = NIPH 512* |
| *Altererythrobacter troitsensis* | *Acinetobacter nosocomialis* |
| *Aminobacter sp. J41* | *Acinetobacter parvus DSM 16617 = CIP 108168* |
| *Anabaena cylindrica* | *Acinetobacter pittii* |
| *Anabaena sp. 39858* | *Acinetobacter radioresistens* |
| *Anabaena sp. 4-3* | *Acinetobacter rudis* |
| *Anabaena sp. CA = ATCC 33047* | *Acinetobacter schindleri CIP 107287* |
| *Anabaena sp. PCC 7108* | *Acinetobacter seifertii* |
| *Anabaena variabilis ATCC 29413* | *Acinetobacter tandoii* |
| *Aquabacterium parvum* | *Acinetobacter towneri* |
| *Aquamicrobium defluvii* | *Acinetobacter ursingii* |
| *Arenimonas malthae* | *Acinetobacter venetianus* |
| *Arenimonas metalli* | *Advenella mimigardefordensis* |
| *Arenimonas sp. SCN 70-307* | *Aeromonas hydrophila* |
| *Azohydromonas australica* | *Aeromonas schubertii* |
| *Azohydromonas lata* | *Aeromonas veronii* |
| *Bacillus cereus group* | *Afipia broomeae* |
| ***Bacillus mycoides*** | *Agrobacterium* |
| *Bacillus thuringiensis str. Al Hakam* | *Agrobacterium fabrum str. C58* |
| *Bacillus weihenstephanensis* | *Agrobacterium radiobacter DSM 30147* |
| *Blastomonas sp. AAP25* | *Agrobacterium rhizogenes* |
| *Bordetella ansorpii* | *Agrobacterium rubi* |
| *Bordetella bronchiseptica* | ***Agrobacterium tumefaciens*** |
| ***Bosea thiooxidans*** | *Alcanivorax nanhaiticus* |
| *Bradyrhizobium sp. BTAi1* | *Aliagarivorans marinus* |
| *Brevundimonas abyssalis TAR-001* | *Aliiglaciecola lipolytica* |
| ***Brevundimonas diminuta*** | *Alkanindiges illinoisensis* |
| *Brevundimonas naejangsanensis* | *Allochromatium vinosum* |
| *Brevundimonas nasdae* | ***Altererythrobacter atlanticus*** |
| *Brevundimonas subvibrioides* | *Aphanocapsa montana BDHKU210001* |
| *Brucella abortus* | *Arcobacter nitrofigilis* |
| *Brucella ceti str. Cudo* | *Asticcacaulis excentricus* |
| *Brucella inopinata* | *Aureimonas frigidaquae* |
| *Brucella melitensis* | *Aureimonas ureilytica* |
| *Brucella microti* | *Azovibrio restrictus* |
| *Brucella neotomae 5K33* | ***Bacillus mycoides*** |
| *Brucella ovis 63/96* | *Beggiatoa alba* |
| *Brucella suis* | *beta proteobacterium AAP99* |
| *Brucella vulpis* | ***Bosea thiooxidans*** |
| *Burkholderia cepacia complex* | *Bradyrhizobium diazoefficiens* |
| *Burkholderia multivorans* | *Bradyrhizobium elkanii* |
| *Burkholderia multivorans ATCC 17616* | *Bradyrhizobium japonicum* |
| *Burkholderia sp. TJI49* | *Bradyrhizobium lupini HPC(L)* |
| *Burkholderia vietnamiensis* | *Bradyrhizobium neotropicale* |
| *Burkholderiaceae* | *Bradyrhizobium oligotrophicum* |
| *Calothrix sp. 336/3* | ***Brevundimonas diminuta*** |
| *Calothrix sp. PCC 7103* | *Burkholderia ubonensis* |
| *Caulobacter crescentus CB15* | *Burkholderiales bacterium GJ-E10* |
| *Caulobacter crescentus NA1000* | *Catenovulum agarivorans* |
| *Caulobacter crescentus OR37* | *Chitinispirillum alkaliphilum* |
| *Caulobacter henricii* | *Chitinivibrio alkaliphilus* |
| *Caulobacter sp. CCH4-E1* | *Colwellia psychrerythraea* |
| *Caulobacter sp. CCH5-E12* | *Comamonas aquatica* |
| *Caulobacter sp. K31* | *Comamonas testosteroni* |
| *Caulobacter vibrioides* | *Congregibacter litoralis* |
| *Cellvibrio japonicus* | ***Croceicoccus naphthovorans*** |
| *Cellvibrio japonicus Ueda107* | *Cupriavidus gilardii* |
| *Chamaesiphon minutus* | ***Cupriavidus taiwanensis*** |
| *Chamaesiphon minutus PCC 6605* | *Cyanobacteria* |
| *Chelativorans sp. BNC1* | *Cycloclasticus zancles* |
| *Chlorogloeopsis fritschii* | *Dechloromonas agitata* |
| *Chroococcidiopsis thermalis* | *Dechloromonas aromatica* |
| *Chroococcidiopsis thermalis PCC 7203* | ***Delftia acidovorans CCUG 15835*** |
| *Chrysosporum ovalisporum* | ***Delftia tsuruhatensis*** |
| *Citromicrobium sp. WPS32* | *Desulfatibacillum alkenivorans* |
| *Comamonas terrae* | *Desulfatitalea tepidiphila* |
| *Corallococcus coralloides* | *Desulfovibrio gigas* |
| *Corallococcus coralloides DSM 2259* | *Desulfurispirillum indicum* |
| ***Croceicoccus naphthovorans*** | *Desulfuromonas acetoxidans* |
| *Cupriavidus basilensis* | *Devosia riboflavina* |
| *Cupriavidus metallidurans* | *Devosia soli* |
| *Cupriavidus metallidurans CH34* | *Ensifer* |
| *Cupriavidus sp. HMR-1* | *Ensifer adhaerens* |
| *Cupriavidus sp. SHE* | *Ensifer sojae* |
| ***Cupriavidus taiwanensis*** | *Ferrimonas balearica* |
| *Curvibacter gracilis* | *Ferrimonas futtsuensis* |
| *Curvibacter lanceolatus* | *Ferrimonas senticii* |
| *Cyanobacterium PCC 7702* | *Geminocystis herdmanii* |
| *Cyanothece sp. PCC 7425* | *Geothrix fermentans* |
| *Cylindrospermum stagnale* | *Gilvimarinus polysaccharolyticus* |
| *Cylindrospermum stagnale PCC 7417* | *Haliea salexigens* |
| *Cystobacter fuscus* | *Herbaspirillum massiliense* |
| *Cystobacter fuscus DSM 2262* | *Herbaspirillum seropedicae* |
| *Dactylococcopsis salina* | *Inquilinus limosus* |
| *Dactylococcopsis salina PCC 8305* | *Kaistia granuli* |
| *Defluviimonas alba* | *Kamptonema* |
| *Delftia* | *Kingella kingae PYKK081* |
| *Delftia acidovorans* | *Lautropia mirabilis* |
| ***Delftia acidovorans CCUG 15835*** | *Luteibacter yeojuensis* |
| *Delftia acidovorans CCUG 274B* | *Lutibaculum baratangense* |
| *Delftia acidovorans SPH-1* | *Lyngbya confervoides BDU141951* |
| ***Delftia tsuruhatensis*** | *Magnetococcus marinus* |
| *Desertifilum sp. IPPAS B-1220* | *Magnetospirillum* |
| *Devosia sp. 17-2-E-8* | *Magnetospirillum caucaseum* |
| *Erythrobacter litoralis HTCC2594* | *Magnetospirillum magneticum* |
| *Erythrobacter sp. EhN03* | *Magnetospirillum sp. XM-1* |
| *Erythrobacter sp. NAP1* | *Marichromatium purpuratum* |
| *Erythrobacter sp. SG61-1L* | *Massilia alkalitolerans* |
| *Ferriphaselus sp. R-1* | *Massilia niastensis* |
| *Filamentous cyanobacterium ESFC-1* | ***Mesorhizobium alhagi*** |
| *Fischerella muscicola* | *Methylobacter* |
| *Fischerella sp. NIES-3754* | *Methylobacter luteus* |
| *Fischerella sp. PCC 9605* | *Methylococcaceae bacterium Sn10-6* |
| *Fortiea contorta* | *Methyloglobulus morosus* |
| *Gallionella capsiferriformans* | *Methylomicrobium album* |
| *Gallionella capsiferriformans ES-2* | *Methylomicrobium buryatense* |
| *gamma proteobacterium HdN1* | *Methylomonas* |
| *Geitlerinema sp. PCC 7105* | *Methylomonas denitrificans* |
| *Geitlerinema sp. PCC 7407* | *Methylomonas methanica* |
| *Gemmobacter nectariphilus* | *Methylophaga thiooxydans* |
| *Gemmobacter sp. LW-1* | *Methylophilus methylotrophus* |
| *Gloeobacter kilaueensis* | *Methylosarcina fibrata* |
| *Gloeobacter kilaueensis JS1* | *Methylosarcina lacus* |
| *Gloeocapsa sp. PCC 7428* | *Methyloversatilis thermotolerans* |
| *Halomonas sp. PBN3* | ***Methyloversatilis universalis*** |
| *Halomonas zincidurans* | *Methylovulum miyakonense* |
| *Halothece sp. PCC 7418* | *Nitrincola nitratireducens* |
| *Herbaspirillum chlorophenolicum* | *Nitrosomonas eutropha* |
| *Hoeflea sp. IMCC20628* | *Nitrososphaera viennensis EN76* |
| *Hyalangium minutum* | *'Nostoc azollae' 0708* |
| *Hyphomicrobium nitrativorans* | *Novispirillum itersonii* |
| *Hyphomicrobium nitrativorans NL23* | ***Novosphingobium aromaticivorans*** |
| *Hyphomonadaceae bacterium BRH_c29* | *Novosphingobium barchaimii* |
| *Hyphomonas* | ***Novosphingobium pentaromativorans*** |
| *Hyphomonas atlantica* | ***Novosphingobium resinovorum*** |
| *Hyphomonas chukchiensis* | ***Novosphingobium sp. SCN 63-17*** |
| *Hyphomonas hirschiana VP5* | *Novosphingobium sp. ST904* |
| *Hyphomonas neptunium ATCC 15444* | ***Novosphingobium tardaugens*** |
| *Hyphomonas polymorpha* | *Oceanobacter kriegii* |
| *Hyphomonas polymorpha PS728* | *Oceanospirillum maris* |
| *Hyphomonas sp. ND6WE1B* | *Ochrobactrum anthropi* |
| *Hyphomonas sp. T16B2* | ***Pannonibacter phragmitetus*** |
| *Ideonella sakaiensis* | ***Paracoccus aminovorans*** |
| *Jannaschia rubra* | ***Paracoccus denitrificans*** |
| *Janthinobacterium sp. CG23_2* | ***Paracoccus versutus*** |
| *Janthinobacterium sp. HH01* | *Parvularcula bermudensis* |
| *Kaistia sp. SCN 65-12* | *Pelagibacterium halotolerans* |
| *Leptolyngbya boryana* | *Photobacterium aquae* |
| *Leptolyngbya sp. Heron Island J* | *Photobacterium ganghwense* |
| *Leptolyngbya sp. JSC-1* | *Photobacterium leiognathi* |
| *Leptolyngbya sp. KIOST-1* | *Photobacterium marinum* |
| *Leptolyngbya sp. NIES-2104* | *Photobacterium profundum* |
| *Leptolyngbya sp. NIES-3755* | *Photobacterium sanctipauli* |
| *Limnoraphis robusta* | *Photobacterium swingsii* |
| *Luteimonas huabeiensis* | *Proteobacteria* |
| *Lyngbya aestuarii* | *Pseudacidovorax intermedius* |
| *Lyngbya sp. PCC 8106* | *Pseudomonas aeruginosa* |
| *Marinobacter manganoxydans MnI7-9* | *Pseudomonas azotifigens* |
| *Massilia sp. LC238* | *Pseudomonas denitrificans* |
| *Mastigocoleus testarum* | *Pseudomonas fluorescens* |
| *Mastigocoleus testarum BC008* | *Pseudomonas fuscovaginae* |
| ***Mesorhizobium alhagi*** | *Pseudomonas knackmussii* |
| *Mesorhizobium metallidurans* | ***Pseudomonas putida*** |
| *Mesorhizobium sp. F7* | *Pseudomonas resinovorans* |
| *Mesorhizobium sp. STM 4661* | ***Pseudomonas stutzeri*** |
| *Methylibium sp. YR605* | ***Pseudoxanthomonas spadix*** |
| *Methylobacterium populi* | *Psychromonas arctica* |
| ***Methyloversatilis universalis*** | *Psychromonas hadalis* |
| *Micavibrio aeruginosavorus* | *Psychromonas ossibalaenae* |
| *Microcoleus sp. PCC 7113* | *Rhizobium alamii* |
| *Microcoleus vaginatus* | *Rhizobium etli* |
| *Microcystis aeruginosa* | *Rhizobium gallicum* |
| *Microcystis panniformis* | *Rhizobium giardinii* |
| *Microcystis sp. T1-4* | *Rhizobium leguminosarum* |
| *Myxococcus fulvus* | *Rhizobium leucaenae* |
| *Myxococcus stipitatus* | *Rhizobium mesoamericanum* |
| *Nitratireductor indicus* | *Rhizobium rhizogenes NBRC 13257* |
| *Nitrobacter winogradskyi* | *Rhizobium rubi NBRC 13261* |
| *Nitrosomonadales bacterium SCN 54-20* | *Rhizobium tropici* |
| *Nitrosospira sp. NpAV* | *Rhodanobacter fulvus* |
| *Nodosilinea nodulosa* | *Rhodanobacter thiooxydans* |
| *Nodularia spumigena* | ***Rhodobacter capsulatus*** |
| *Nodularia spumigena CENA596* | *Rhodocyclaceae bacterium Paddy-1* |
| *Nostoc punctiforme* | ***Rhodopseudomonas palustris*** |
| *Nostoc sp. KVJ20* | *Rubrivivax gelatinosus* |
| *Nostoc sp. MBR 210* | *Sedimenticola thiotaurini* |
| *Nostoc sp. NIES-3756* | *Serratia marcescens* |
| *Nostoc sp. PCC 7107* | *Serratia odorífera* |
| *Nostoc sp. PCC 7120* | *Shewanella amazonensis* |
| *Nostoc sp. PCC 7524* | *Shewanella colwelliana* |
| ***Novosphingobium aromaticivorans*** | *Shewanella denitrificans* |
| *Novosphingobium lentum* | *Shewanella loihica* |
| *Novosphingobium lindaniclasticum* | *Shewanella mangrovi* |
| *Novosphingobium nitrogenifigens* | *Simiduia agarivorans* |
| ***Novosphingobium pentaromativorans*** | *Simplicispira psychrophila* |
| ***Novosphingobium resinovorum*** | *Sinorhizobium* |
| *Novosphingobium sp. MBES04* | *Sinorhizobium fredii* |
| *Novosphingobium sp. PP1Y* | *Sinorhizobium medicae WSM419* |
| ***Novosphingobium sp. SCN 63-17*** | *Sinorhizobium meliloti* |
| *Novosphingobium sp. SCN 66-18* | ***Sphingobium baderi LL03*** |
| *Novosphingobium subterraneum* | *Sphingomonadaceae* |
| ***Novosphingobium tardaugens*** | *Sphingomonas* |
| *Oceanibulbus sp. HI0027* | *Sphingomonas changbaiensis* |
| *Oligotropha carboxidovorans* | *Sphingomonas elodea* |
| *Oscillatoria acuminata* | *Sphingomonas hengshuiensis* |
| *Oscillatoria nigro-viridis* | ***Sphingomonas melonis*** |
| *Oscillatoriales cyanobacterium MTP1* | *Sphingomonas paucimobilis NBRC 13935* |
| *Oxalobacteraceae bacterium AB_14* | *Sphingomonas sp. S17* |
| *Paenirhodobacter sp. MME-103* | *Sphingomonas wittichii* |
| ***Pannonibacter phragmitetus*** | ***Sphingopyxis macrogoltabida*** |
| ***Paracoccus aminovorans*** | *Sphingopyxis terrae* |
| ***Paracoccus denitrificans*** | *Spongiibacter tropicus* |
| *Paracoccus halophilus* | *Stanieria cyanosphaera* |
| *Paracoccus pantotrophus* | ***Stenotrophomonas acidaminiphila*** |
| *Paracoccus sanguinis* | *Stenotrophomonas humi* |
| *Paracoccus sp. J55* | ***Stenotrophomonas maltophilia*** |
| *Paracoccus sp. MKU1* | *Stenotrophomonas panacihumi* |
| *Paracoccus sp. N5* | ***Stenotrophomonas rhizophila*** |
| ***Paracoccus versutus*** | *Sulfuricurvum kujiense* |
| *Paracoccus yeei* | *Tepidicaulis marinus* |
| *Parvibaculum lavamentivorans* | *Teredinibacter turnerae* |
| *Pelagibacterium sp. SCN 63-126* | *Thalassolituus oleivorans* |
| *Phaeobacter sp. CECT 5382* | *Thauera* |
| *Phenylobacterium sp. SCN 70-31* | *Thauera terpenica 58Eu* |
| *Phenylobacterium zucineum* | *Thermomonas fusca* |
| *Phormidesmis priestleyi* | *Thiocapsa marina* |
| *Phormidium sp. OSCR* | *Tistrella mobilis* |
| *Phormidium willei* | *Trichormus azollae* |
| *Polymorphum gilvum* | *Uliginosibacterium gangwonense* |
| *Porphyrobacter cryptus* | ***Verrucomicrobiae bacterium DG1235*** |
| *Prosthecomicrobium hirschii* | *Vibrio campbellii* |
| *Pseudanabaena sp. 'Roaring Creek'* | *Vibrio coralliilyticus* |
| *Pseudochrobactrum sp. AO18b* | *Vibrio crassostreae* |
| *Pseudomonas bauzanensis* | *Vibrio diazotrophicus* |
| *Pseudomonas caeni* | *Vibrio fluvialis* |
| *Pseudomonas koreensis* | *Vibrio mediterranei* |
| *Pseudomonas lundensis* | *Vibrio parahaemolyticus* |
| *Pseudomonas monteilii* | *Vibrio proteolyticus* |
| *Pseudomonas mosselii* | *Vibrio tasmaniensis* |
| *Pseudomonas nitroreducens* | *Vibrio vulnificus* |
| *Pseudomonas parafulva* | *Xanthomonas albilineans* |
| *Pseudomonas plecoglossicida* | *Xanthomonas arboricola* |
| ***Pseudomonas putida*** | *Xanthomonas campestris* |
| *Pseudomonas sp. 10-1B* | *Xanthomonas maliensis* |
| *Pseudomonas sp. ABFPK* | *Xanthomonas sacchari* |
| *Pseudomonas sp. C5pp* | *Xenophilus azovorans* |
| *Pseudomonas sp. CF150* |  |
| *Pseudomonas sp. ICMP 19500* |  |
| *Pseudomonas sp. JY-Q* |  |
| *Pseudomonas sp. NBRC 111120* |  |
| *Pseudomonas sp. Root9* |  |
| *Pseudomonas sp. TJI-51* |  |
| *Pseudomonas sp. TKP* |  |
| *Pseudomonas sp. URMO17WK12:I11* |  |
| *Pseudomonas sp. VLB120* |  |
| ***Pseudomonas stutzeri*** |  |
| *Pseudomonas synxantha* |  |
| *Pseudomonas taiwanensis SJ9* |  |
| *Pseudorhodobacter aquimaris* |  |
| *Pseudorhodoferax sp. Leaf267* |  |
| *Pseudoruegeria sabulilitoris* |  |
| *Pseudoxanthomonas mexicana* |  |
| *Pseudoxanthomonas sp. Root630* |  |
| ***Pseudoxanthomonas spadix*** |  |
| *Ralstonia pickettii 12J* |  |
| *Rheinheimera nanhaiensis* |  |
| *Rhizobiales bacterium CCH3-A5* |  |
| ***Rhodobacter capsulatus*** |  |
| *Rhodobacter sphaeroides* |  |
| *Rhodobacteraceae bacterium HLUCCA12* |  |
| *Rhodocyclaceae bacterium PG1-Ca6* |  |
| ***Rhodopseudomonas palustris*** |  |
| *Rhodopseudomonas sp. AAP120* |  |
| *Rhodovulum sulfidophilum* |  |
| *Richelia intracellularis* |  |
| *Robiginitomaculum antarcticum* |  |
| *Scytonema hofmannii* |  |
| *Scytonema tolypothrichoides* |  |
| *Shinella* |  |
| ***Sphingobium baderi LL03*** |  |
| *Sphingobium chinhatense IP26* |  |
| *Sphingobium chlorophenolicum* |  |
| *Sphingobium chungbukense* |  |
| *Sphingobium cloacae* |  |
| *Sphingobium czechense* |  |
| *Sphingobium herbicidovorans NBRC 16415* |  |
| *Sphingobium japonicum UT26S* |  |
| *Sphingobium lactosutens DS20* |  |
| *Sphingobium lucknowense F2* |  |
| *Sphingobium quisquiliarum P25* |  |
| *Sphingobium yanoikuyae* |  |
| *Sphingomonadales bacterium EhC05* |  |
| *Sphingomonas adhaesiva* |  |
| ***Sphingomonas melonis*** |  |
| *Sphingomonas parapaucimobilis NBRC 15100* |  |
| *Sphingomonas paucimobilis* |  |
| *Sphingomonas pituitosa* |  |
| *Sphingomonas sanguinis* |  |
| *Sphingomonas sanxanigenens DSM 19645 = NX02* |  |
| *Sphingomonas taxi* |  |
| *Sphingopyxis alaskensis* |  |
| *Sphingopyxis fribergensis* |  |
| *Sphingopyxis granuli* |  |
| ***Sphingopyxis macrogoltabida*** |  |
| *Sphingopyxis terrae NBRC 15098* |  |
| *Spongiibacter sp. IMCC21906* |  |
| ***Stenotrophomonas acidaminiphila*** |  |
| ***Stenotrophomonas maltophilia*** |  |
| *Stenotrophomonas nitritireducens* |  |
| ***Stenotrophomonas rhizophila*** |  |
| *Stenotrophomonas sp. KCTC 12332* |  |
| *Stenotrophomonas sp. Leaf70* |  |
| *Sulfitobacter sp. NAS-14.1* |  |
| *Synechococcus sp. PCC 7335* |  |
| *Synechocystis sp. PCC 6803* |  |
| *Thalassospira sp. Nap_22* |  |
| *Thermithiobacillus tepidarius* |  |
| *Thioalkalivibrio sulfidiphilus* |  |
| *Thiobacillus sp. SCN 63-1177* |  |
| *Thioclava atlántica* |  |
| *Tolypothrix bouteillei* |  |
| *Tolypothrix campylonemoides* |  |
| *Tolypothrix sp. PCC 7601* |  |
| *Variovorax paradoxus* |  |
| ***Verrucomicrobiae bacterium DG1235*** |  |
| *Xanthobacter autotrophicus* |  |
| *Xanthomonadaceae bacterium SCN 69-48* |  |
| *Xylophilus ampelinus* |  |
|  |  |
