# Supplementary figures and images for: Defining the Roles of the Cation Diffusion Facilitators in Fe2+/Zn2+ Homeostasis and Establishment of Their Participation in Virulence in Pseudomonas aeruginosa
Source: Front Cell Infect Microbiol. 2017 Mar 20;7:84. doi: 10.3389/fcimb.2017.00084 (PMC5357649; doi:10.3389/fcimb.2017.00084)

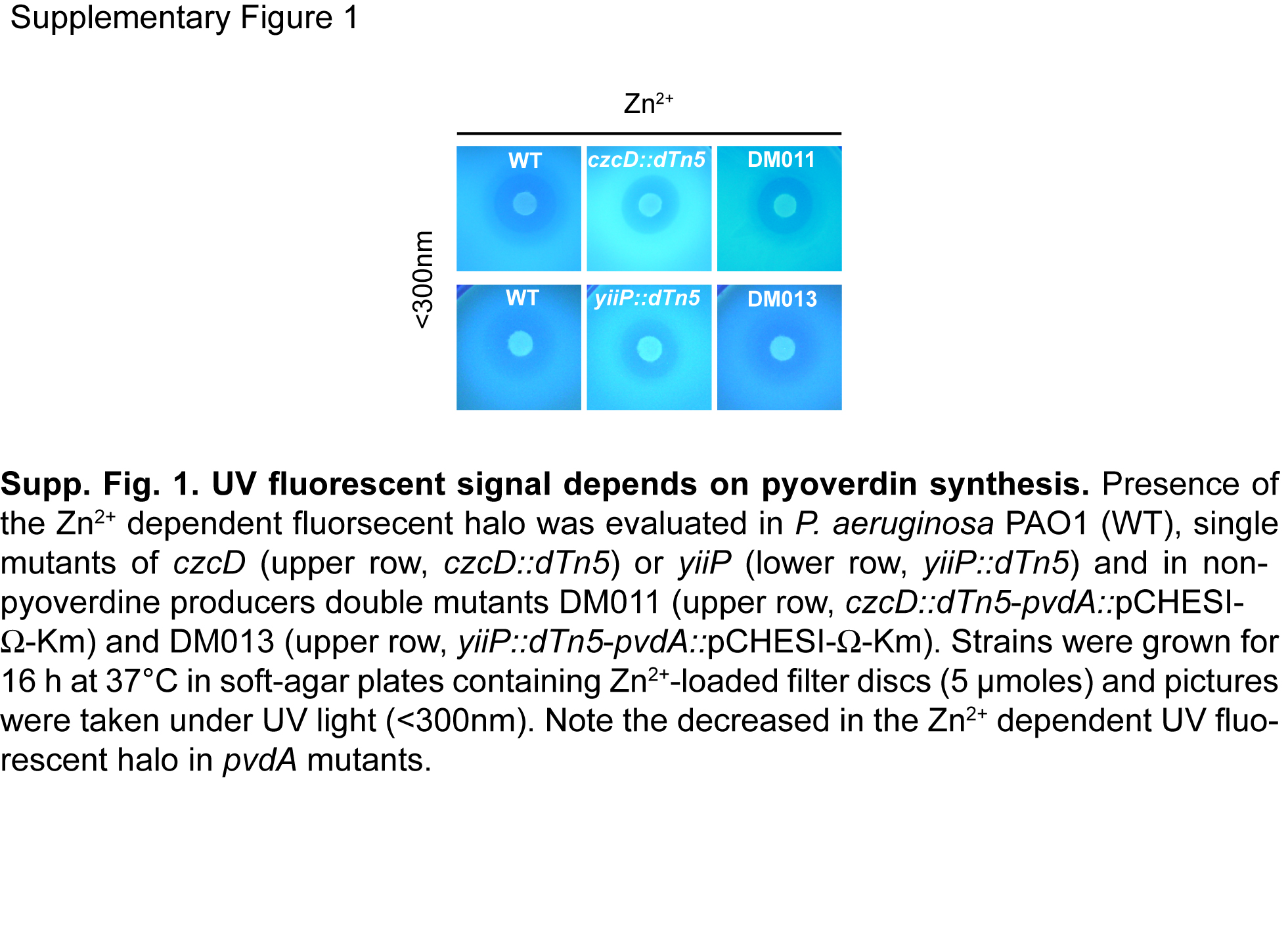

Supplement: Supplementary file 2 [file Image1.JPEG]
